# The Effects of Myo-Inositol and B and D Vitamin Supplementation in the db/+ Mouse Model of Gestational Diabetes Mellitus

**DOI:** 10.3390/nu9020141

**Published:** 2017-02-15

**Authors:** Jasmine F. Plows, Florence Budin, Rebecka A. M. Andersson, Valerie J. Mills, Katherine Mace, Sandra T. Davidge, Mark H. Vickers, Philip N. Baker, Irma Silva-Zolezzi, Joanna L. Stanley

**Affiliations:** 1 Liggins Institute, University of Auckland, Auckland 1021, New Zealand; jasmine.plows@auckland.ac.nz (J.F.P.); reban468@student.liu.se (R.A.M.A.); valeriejeanmills@gmail.com (V.J.M.); m.vickers@auckland.ac.nz (M.H.V.); philip.baker@leicester.ac.uk (P.N.B.); 2 Nestle Research Center, Lausanne 1000, Switzerland; florence.budin@rdls.nestle.com (F.B.); catherine.mace@rdls.nestle.com (K.M.); irma.silvazolezzi@rdls.nestle.com (I.S.-Z.); 3 University of Alberta, Edmonton, AB T6G 2R3, Canada; sandra.davidge@ualberta.ca; 4 College of Medicine, Biological Sciences and Psychology, University of Leicester, Leicester LE1 7RH, UK

**Keywords:** gestational diabetes, myo-inositol, B2, B6, B12, D, Riboflavin, db/+, db/db, mice, LepR

## Abstract

Gestational diabetes mellitus (GDM) is a growing concern, affecting an increasing number of pregnant women worldwide. By predisposing both the affected mothers and children to future disease, GDM contributes to an intergenerational cycle of obesity and diabetes. In order to stop this cycle, safe and effective treatments for GDM are required. This study sought to determine the treatment effects of dietary supplementation with myo-inositol (MI) and vitamins B2, B6, B12, and D in a mouse model of GDM (pregnant db/+ dams). In addition, the individual effects of vitamin B2 were examined. Suboptimal B2 increased body weight and fat deposition, decreased GLUT4 adipose tissue expression, and increased expression of inflammatory markers. MI supplementation reduced weight and fat deposition, and reduced expression of inflammatory markers in adipose tissue of mice on suboptimal B2. MI also significantly reduced the hyperleptinemia observed in db/+ mice, when combined with supplemented B2. MI was generally associated with adipose tissue markers of improved insulin sensitivity and glucose uptake, while the combination of vitamins B2, B6, B12, and D was associated with a reduction in adipose inflammatory marker expression. These results suggest that supplementation with MI and vitamin B2 could be beneficial for the treatment/prevention of GDM.

## 1. Introduction

Gestational diabetes mellitus (GDM) is a serious pregnancy complication, characterised by glucose intolerance that either develops or is first recognised during pregnancy [1]. The prevalence of GDM has rapidly increased in recent years, in parallel with the obesity, cardiovascular disease, and type 2 diabetes epidemics [2]. It now affects approximately 10% of pregnancies in the United States and up to 20% of pregnancies in some countries, such as India and China [3,4,5]. GDM is a major concern because of the various short- and long-term health consequences it poses for both the mother and the child. Women with GDM are more likely to experience further pregnancy complications, such as pre-eclampsia [6], and to develop type-2 diabetes (T2DM) later in life [7,8]. Babies born from pregnancies complicated by GDM are more likely to be born large for gestational age (LGA), and to be affected by obesity and type 2 diabetes in the future [9]. Animal studies have revealed that these effects can persist beyond one generation and into the next, resulting in an inter-generational cycle of disease [10,11]. Therefore, prevention of GDM could be beneficial for improving the health of the population as a whole.

There are limited treatment options currently available to women with GDM. Insulin is the mainstay of treatment, but has a number of caveats attached to its use. For example, insulin is associated with hypoglycaemia and increased weight gain during pregnancy, which are themselves linked to adverse pregnancy outcomes, and it requires self-injection [12]. Metformin is an effective treatment for GDM and other disorders of insulin resistance, but often requires additional treatment with insulin in order to maintain adequate glycaemic control [13]. Metformin is also known to cross the placenta, and the long-term effects on the unborn child are still unknown [14]. In addition, metformin has some side-effects, such as gastrointestinal discomfort [15,16], and has been associated with depleted vitamin B12 status [17]. Another emerging treatment, sulfonylurea glyburide, carries similar unknowns [18,19]. For these reasons, much research is dedicated to the development of alternative treatments.

The current study sought to evaluate the effectiveness of myo-inositol (MI) and B and D vitamin supplementation, taken both before and during pregnancy, for the prevention/treatment of GDM. MI is a carbohydrate consumed and produced within the body that is a precursor for various phosphorylated derivatives such as phosphatidylinositol triphosphate (PIP)—a downstream effector of insulin signalling. MI therefore acts as an insulin-sensitizing agent, and has shown promise as a treatment for diseases of glucose intolerance, such as polycystic ovarian syndrome (PCOS) and T2DM [20,21,22]. There is evidence that MI supplementation also improves glucose tolerance during pregnancy, and therefore could be an effective treatment for GDM [23,24].

In addition, recent cross-sectional and observational studies have noted vitamin deficiencies in individuals with GDM and/or associated conditions, including vitamins B2 [25,26,27], B6 [28,29,30], B12 [31,32,33] and D [34,35,36,37]. This has led to the hypothesis that supplementation with these agents during pregnancy could improve maternal health and long-term outcomes for the child. Vitamin B2 acts as a cofactor in many metabolic reactions, including the electron transport chain, the citric acid cycle, and fatty acid catabolism [38]. The combination of vitamins B2, B6, and B12 also plays an important role in homocysteine homeostasis. High concentrations of homocysteine are strongly correlated with GDM and related pathologies [39,40,41,42,43]. These vitamins are also essential for one-carbon metabolism and DNA methylation, which drive epigenetic programming of the foetus during pregnancy, and therefore may influence the long-term health of the child (for a review on this topic, see [44]). While vitamins B6, B12, and D have already shown promise as potential treatments for GDM and associated diseases during pregnancy [45,46,47,48,49,50,51], vitamin B2 has not been investigated as thoroughly in this area. This is despite the fact that B2 has been shown to protect against inflammation-induced cell damage and the development of insulin resistance in the non-pregnant state [52,53,54]. For this reason, the current study examined the individual role of vitamin B2 in GDM by comparing groups of mice receiving suboptimal, normal, and supplemented levels. Further, an ongoing clinical trial is evaluating the effects of a nutritional supplement, taken pre-conception and during pregnancy, on maternal glucose tolerance [55]. The supplement includes, amongst other micronutrients, MI and vitamins B2, B6, B12, and D. Therefore, we also included a “vitamin mix” group in the current study, to allow for a more complete comparison of results between the pre-clinical and clinical studies.

The overall aim of this study was to determine the effects of MI and B and D vitamins, both individually and in combination, in a mouse model of GDM. The model used was the db/+ mouse, which is heterozygous for a point mutation of the leptin receptor. These mice spontaneously develop hyperphagia, hyperleptinemia, increased weight and fat deposition, and glucose intolerance during, but not prior to, pregnancy [56,57,58,59].

We hypothesised that dietary supplementation with MI, vitamin B2, and vitamins B6, 12, and D, would improve glucose tolerance and other markers of GDM in db/+ mice, with the most profound effect in the group combining them all. We also hypothesised that a diet suboptimal in vitamin B2 would worsen metabolic health, and that the addition of MI would negate this phenotype.

## 2. Materials and Methods

### 2.1. Animals and Genotyping

All animal procedures were approved by the AgResearch Animal Ethics Committees in accordance with the recommendations of the New Zealand Animal Welfare Act, 1999. Animals were housed in a 12-h light, 12-h dark cycle at 22 °C, with 40%–45% humidity and free access to food and water at all times. LepR^db/+^ (db/+) mice (strain B6.BKS(D)-Leprdb/+/J; stock number 000697) were obtained from the Jackson Laboratory, Bar Harbor, ME, USA, and housed at the small animal containment unit at AgResearch, Waikato, New Zealand. 

Male and female db/+ mice were mated, and tail clippings were taken from weanlings at three weeks of age for genotyping, as detailed in the Supplementary Materials. Wild-type (WT) mice were used as controls, while heterozygous (db/+) mice were used as experimental animals. Homozygotes were not used in the study, as they are infertile.

### 2.2. Experimental Groups

All mice were weaned on to AIN-93G (Research Diets Inc., New Brunswick, NJ, USA) at three weeks of age. At four weeks of age, after genotyping was complete, WT and db/+ female offspring were separated and randomized to one of eight diet groups (Table 1). Levels of micronutrients were derived based on previous micronutrient deficiency studies performed in mice [60,61,62]. The amount of folic acid was kept constant across all groups in the AIN-93G diet (2 mg/kg diet), which is specially formulated for gestating rodents. Mice were kept on experimental diets for eight weeks before mating commenced. Mice were mated with males of the same genotype, and pregnancy was confirmed by the existence of a vaginal plug, which was denoted day 0.5 of gestation (GD0.5). Pregnant females were singly-housed, and weighed at GD0.5 and GD18.5. They were kept on their assigned diet throughout gestation, and food intake was measured over the course of the pregnancy by subtracting the amount of food left in grams at GD18.5 from the amount of food added in grams at GD0.5. Seven to twelve mice completed the study in each group, for a total of 143 mice overall. 

### 2.3. Oral Glucose Tolerance Test

Glucose tolerance was measured at GD16.5. Following a 6 h fast, mice were given a 2 g/kg glucose solution via oral gavage [63]. Blood was sampled from the tail tip at 0, 30, 60, 90, and 120 min and glucose was measured using an Optimum FreeStyle Glucometer (Abbott Diabetes Care, Oxfordshire, UK).

### 2.4. Tissue Collection

At GD18.5, females were fasted for 6 h, at which point weight and fasting glucose were measured. A tail blood sample was collected in an EDTA (ethylenediaminetetraacetic acid)-coated tube for later analysis of fasting insulin and leptin concentrations. Mice were then culled via cervical dislocation, and further blood was collected via cardiac puncture. The uterus was removed and placed in a solution of ice-cold saline. The pancreas, spleen, retroperitoneal fat, gonadal fat, perirenal fat, kidneys, and liver were weighed and snap frozen for future analysis. Each foetus and its individual placenta were then removed from the uterus and weighed, and foetal crown-rump and abdominal lengths were measured. Placentae and foetal livers were snap frozen, and foetal tails were also collected for genotyping.

### 2.5. Plasma Assays

Plasma was obtained by centrifuging blood taken at GD18.5 for 10 min at 10,000 rpm. Plasma was then stored at −80 °C. UltraSensitive Mouse Insulin ELISA (#90080) and Mouse Leptin ELISA (#90030) kits (Crystal Chem., Downers Grove, IL, USA) were used according to the manufacturer’s instructions. Insulin resistance score was determined using HOMA-IR, which was calculated as follows:

HOMA-IR = (fasting glucose (mmol/L) × fasting insulin (mU/L))/22.5

### 2.6. Gene Expression Analysis

RNA was extracted from gonadal fat samples by homogenizing in TRIzol (Thermo Fisher Scientific, Waltham, MA, USA). cDNA was produced using High-Capacity cDNA Reverse Transcription Kit (Thermo Fisher Scientific, Waltham, MA, USA). Gene expression analysis was performed using Taqman Fast Advanced Master Mix and pre-designed Taqman probes for 16 genes involved in the pathogenesis of GDM and related diseases (Akt2, Igf1R, IRS1, GLUT4, Acsl1, Ccr5, LepR, Tlr4, Fas, Tnfrsf1b, Gys1, Gck, Pck1, G6pc, Il-1b, and Nlrp3). Fluorescence was assessed using RT-PCR (Bio-Rad Laboratories, Hercules, CA, USA), and CT values were normalised to a housekeeper gene (18S). All gene expression results were subsequently converted to expression fold change relative to the control group (db/+ mice on AIN-93G diet: normal B2 with no added MI).

### 2.7. Statistics

All statistical analysis was performed by IBM SPSS (Armonk, NY, USA). Graphs were produced using GraphPad Prism. Statistical tests were designed to compare the effects of genotype (WT vs. db/+), the effects of MI status (no added MI vs. added MI), and the effects of vitamin status (suboptimal B2, normal B2, supplemented B2, and vitamin mix) via three-way ANOVA. To check if the data were normally distributed, the Shapiro–Wilk’s test of normality was performed. Levene’s test for equality of variances was used in order to test homogeneity of variances. In the case of failure of one or both of these tests, data were appropriately transformed to achieve normal distribution. If the same results were obtained with unadjusted and transformed data, the unadjusted data were used. Data were first assessed for a three-way interaction. Where a three-way interaction was present, this was followed by testing for a simple two-way interaction within both WT and db/+ mice, and then further determining simple-simple main effects and simple-simple comparisons. If a three-way interaction was not present, it was established whether any significant two-way interaction existed. If a two-way interaction existed, the simple main effect and simple comparisons of the independent variable of interest was assessed. If neither a two-way nor three-way interaction was present, it was established whether a main effect of one of the independent variables existed. Statistical significance was accepted at the *p* < 0.05 level, and a Bonferroni correction was applied to all simple and simple-simple tests. Data are presented as mean ± the standard error of the mean, unless otherwise stated.

## 3. Results

### 3.1. Body Weight at GD0.5 and GD18.5

#### 3.1.1. Weight at the Beginning of Pregnancy (GD0.5)

**Figure 1:**
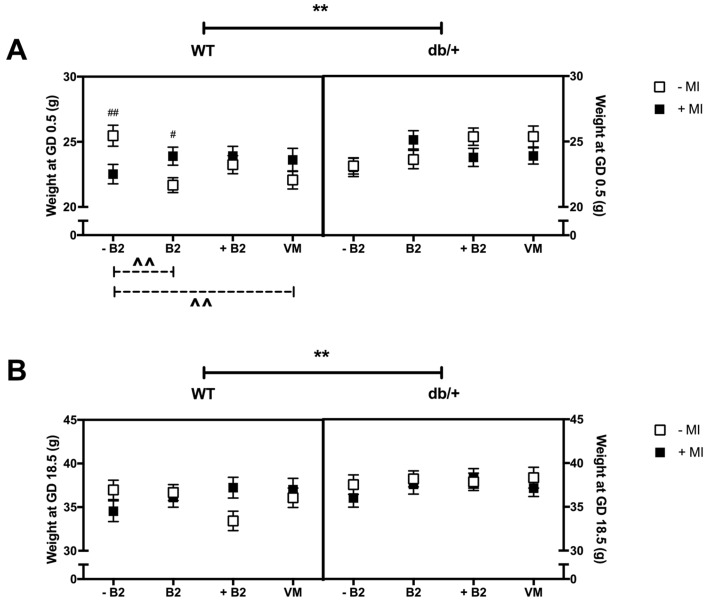
(**A**) Body weight at GD0.5 (g). * indicates a main effect; # indicates a difference between groups with and without MI.; and ^ indicates a difference between vitamin status groups, where a dashed line indicates the difference occurs in the group without MI, and a solid line indicates that the difference occurs in the group with MI. There was a significant difference between WT and db/+ overall—indicated by ** (*p* = 0.017). There was also a significant difference between WT mice with and without MI in the –B2 (*p* = 0.008) and B2 (*p* = 0.015) groups—indicated by #. In addition, there was a significant difference between WT mice with suboptimal and normal B2 in the absence of MI (indicated by ^^ (*p* = 0.001)), and between WT with suboptimal and vitamin mix in the absence of MI (indicated by ^^ (*p* = 0.011)); (**B**) Body weight at GD18.5 (g). There was a significant difference between WT and db/+ overall—indicated by ** (*p* = 0.004). *n* = 7–12 mice per group.

The db/+ mice were heavier than WT mice at the beginning of pregnancy (*p* = 0.017; Figure 1A). In addition, there was a three-way interaction between genotype, MI, and B vitamin status on weight at the beginning of pregnancy (*p* = 0.03), as a result of a simple two-way interaction between MI and B vitamin status in WT mice (*p* = 0.004).

*Effect of myo-inositol status*: Within the suboptimal B2 group, WT mice supplemented with MI weighed less than mice without MI (*p* = 0.008). However, in the normal B2 group, WT mice supplemented with MI weighed more than mice without MI (*p* = 0.015).

*Effect of vitamin status*: In the absence of MI, suboptimal B2 was associated with increased body weight, compared to normal B2 (*p* = 0.001) and vitamin mix (*p* = 0.011). This was observed in WT mice only.

#### 3.1.2. Weight at GD18.5

The db/+ mice were heavier than WT mice at the end of pregnancy (*p* = 0.004; Figure 1B). There were no significant interactions or differences between any of the groups on weight at GD18.5.

### 3.2. Pregnancy Weight Gain, Food Intake, and Litter Size

#### 3.2.1. Weight Gain over Pregnancy

**Figure 2:**
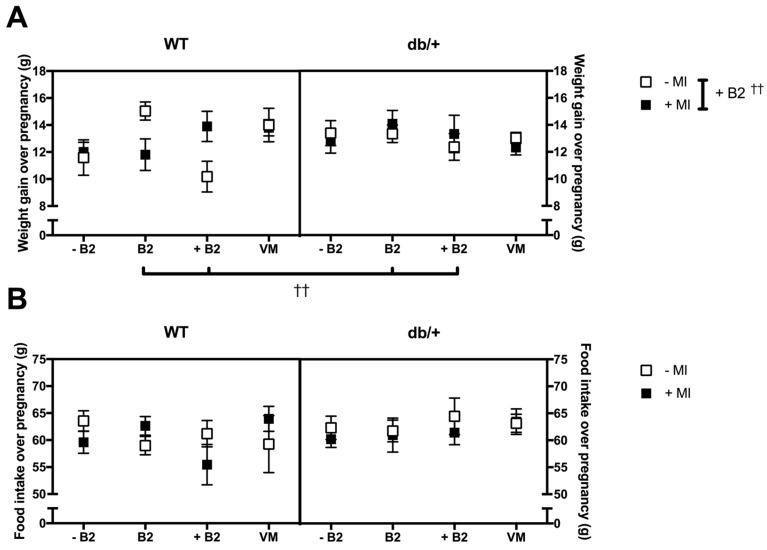

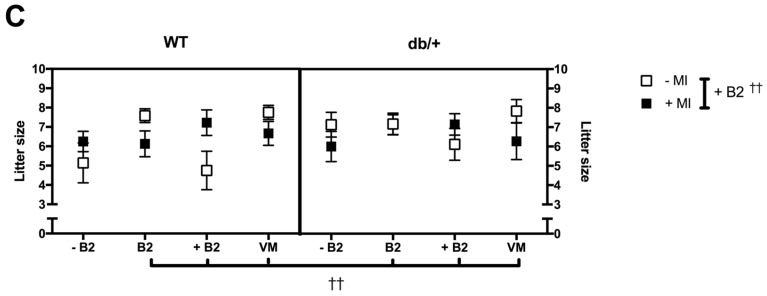
(**A**) Weight gain over pregnancy (g). ^†^ indicates a simple main effect. There was a significant difference between mice on normal B2 and supplemented B2, in the absence of MI, as indicated by ^††^ under the graph. There was a significant difference between mice with and without MI in the presence of supplemented B2, as indicated by †† on the right of the key (*p* = 0.015); (**B**) Food intake over pregnancy (g). There was no effect of genotype, MI, or vitamin status on food intake over pregnancy; (**C**) Litter size at GD18.5. Mice supplemented with B2 had significantly fewer foetuses per litter than mice in the normal B2 and vitamin mix groups, as indicated by †† under the graph. Mice supplemented with MI had more foetuses per litter than mice not receiving MI in the supplemented B2 group, as indicated by ^††^ on the right of the key (*p* = 0.016). These differences generally match the differences seen in weight gain (Figure 2A), indicating that litter size was the primary driver of weight gain over pregnancy. *n* = 7–12 mice per group.

There was no significant three-way interaction between genotype, MI, and vitamin status (*p* = 0.075; Figure 2A). However, there was a significant two-way interaction between MI and vitamin status (*p* = 0.049).

*Effect of myo-inositol status*: MI had an effect only in the supplemented B2 group. Independent of genotype, mice supplemented with MI gained more weight over pregnancy than mice without MI, in the supplemented B2 group (*p* = 0.015). 

*Effect of vitamin status*: B2 had an effect only in the absence of MI. Ignoring genotype, mice in the supplemented B2 group gained less weight over pregnancy than mice in the normal B2 group (*p* = 0.006).

#### 3.2.2. Food Intake over Pregnancy

There were no main effects or interactions between any of the groups on food intake over pregnancy (Figure 2B).

#### 3.2.3. Litter Size

There was no significant three-way interaction between genotype, MI, and vitamin status on litter size (*p* = 0.273; Figure 2C). However, there was a significant two-way interaction between MI and vitamin status (*p* = 0.019).

*Effect of myo-inositol status*: MI had an effect on litter size only in the supplemented B2 group. Mice supplemented with MI had a larger litter size than mice not receiving MI (*p* = 0.016).

*Effect of vitamin status*: An effect of vitamin B2 was only seen in non-MI supplemented groups. Mice on supplemented B2 had a smaller litter size than mice in the normal B2 (*p* = 0.020) and vitamin mix groups (*p* = 0.011).

*Relation to weight gain over pregnancy*—The effects of litter size generally matched the pattern of weight gain over pregnancy, indicating that the differences in weight gain seen were likely due to differences in litter size.

### 3.3. Glucose Tolerance during Pregnancy

A three-way ANOVA was performed for each time point of the oral glucose tolerance test (0, 30, 60, 90, and 120 min), and for the overall area under the OGTT curve (AUC), in order to assess if there were any effects of treatment.

Differences in glucose tolerance between WT and db/+ mice during pregnancy had been expected, however, no such differences were identified (see Figures S1 and S2).

**Figure 3:**
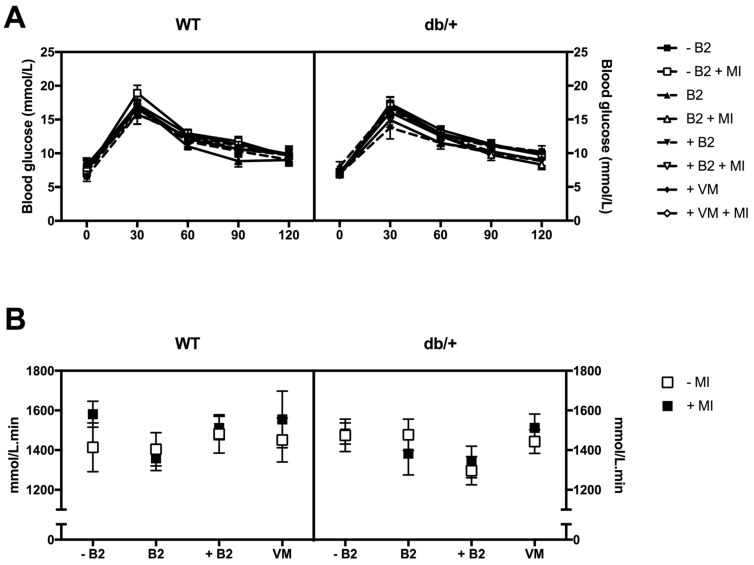
(**A**) Glucose tolerance at GD16.5, OGTT for all treatment groups in both WT and db/+ mice; and (**B**) area under the curve of the OGTT plots at GD16.5. *n* = 7–12 mice per group.

There were no main effects or interactions between any of the groups at any of the time points of the OGTT (Figure 3A). There were also no main effects or interactions between any of the groups on area under the curve of the OGTT (Figure 3B).

### 3.4. Fasting Plasma Glucose, Insulin, HOMA-IR, and Leptin at Day 18.5 of Pregnancy

**Figure 4:**
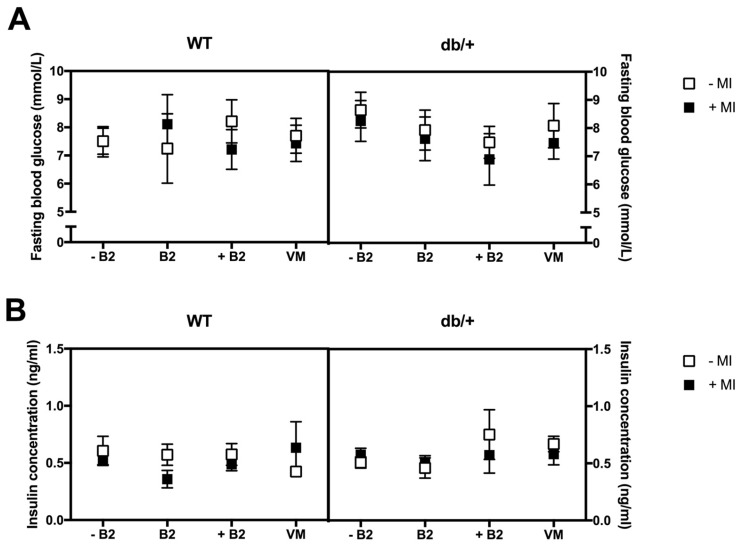

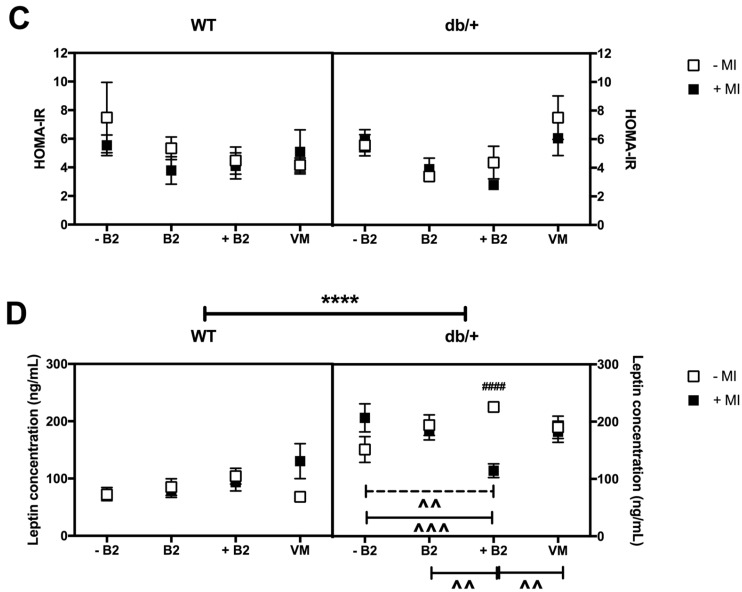
No changes were observed in: (**A**) fsting glucose; (**B**) fasting insulin; (**C**) HOMA-IR index at GD18.5; and (**D**) fasting plasma leptin at GD18.5. * indicates a main effect; ^#^ indicates a difference between groups with and without MI; and ^ indicates a difference between vitamin status groups, where a dashed line indicates the difference occurs in the group without MI, and a solid line indicates that the difference occurs in the group with MI. db/+ mice had higher fasting plasma leptin concentrations than WT mice, as indicated by **** (*p* < 0.0001). There was a significant effect of MI supplementation in db/+ mice on supplemented B2, as indicated by ^####^ (*p* < 0.0001). There was also a significant difference between mice on supplemented B2 and the suboptimal B2 group, in the absence of MI—indicated by ^^ (*p* = 0.007). The opposite effect was observed in the presence of MI—indicated by ^^^ (*p* = 0.001). Mice in the supplemented B2 group also had lower leptin than mice in the normal B2 (*p* = 0.014) and vitamin mix (*p* = 0.010) groups in the presence of MI—indicated by the ^^ under the graph. *n* = 7–12 mice per group.

There were no significant main effects or interactions between any of the groups on fasting blood glucose (Figure 4A), fasting insulin (Figure 4B), or HOMA-IR (Figure 4C).

The db/+ mice had significantly higher fasting plasma leptin at GD18.5 than WT mice (*p* < 0.0001; Figure 4D).

There was a three-way interaction between genotype, MI, and B2 (*p* = 0.005), as a result of a simple two-way interaction between MI and B2, in db/+ mice (*p* = 0.0001).

*Effect of myo-inositol status*: MI supplementation significantly decreased leptin concentration in db/+ mice on supplemented B2 (*p* < 0.0001).

*Effect of vitamin status*: Mice in the supplemented B2 group had higher leptin than those in the suboptimal B2 group, in the absence of MI (*p* = 0.007). However, this was reversed in the presence of MI: mice in the supplemented B2 group had lower leptin than those mice in the suboptimal B2 group (*p* = 0.001). In addition, mice in the supplemented B2 group had lower leptin concentrations than mice in both the normal B2 (*p* = 0.014) and vitamin mix groups (*p* = 0.010), but only in the presence of MI.

### 3.5. Weights of Major Organs at Day 18.5 of Pregnancy

There were no main effects or interactions between any of the groups on the weights of any of the major organs in this study (Table S2).

### 3.6. Fat Distribution at Day 18.5 of Pregnancy

#### 3.6.1. Retroperitoneal Fat

**Figure 5:**
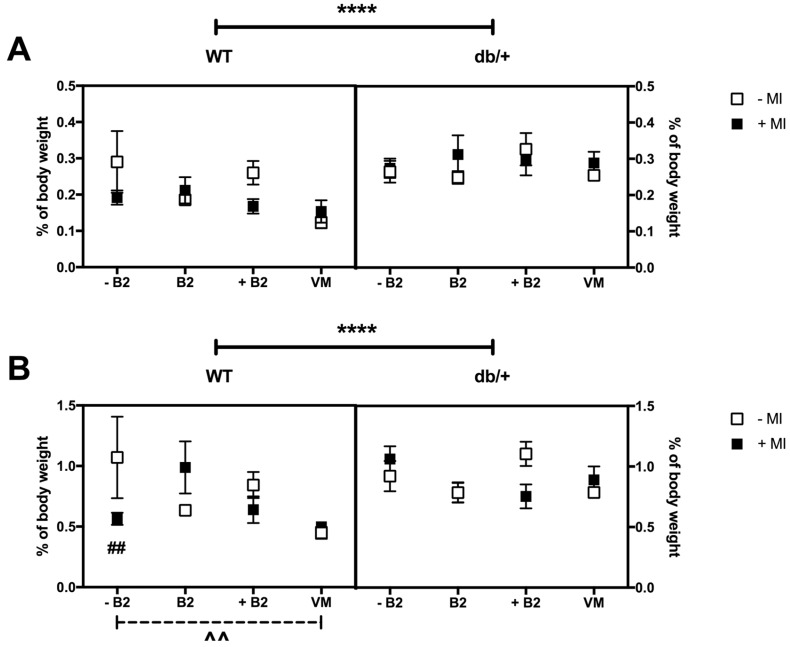

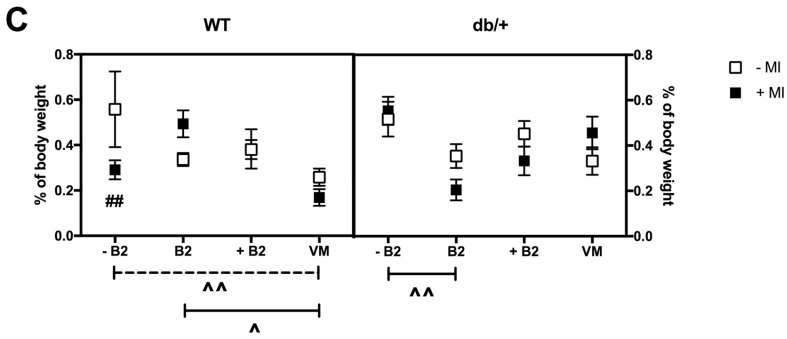
(**A**) Retroperitoneal fat deposition at GD18.5 expressed as percentage of total body weight. * indicates a main effect. db/+ mice had significantly more retroperitoneal fat than WT mice, as indicated by **** (*p* < 0.0001); (**B**) Gonadal fat deposition at GD18.5 expressed as percentage of total body weight. ^#^ indicates a difference between groups with and without MI; and ^ indicates a difference between vitamin status groups, where a dashed line indicates the difference occurs in the group without MI, and a solid line indicates that the difference occurs in the group with MI. db/+ mice had significantly more gonadal fat than WT mice, as indicated by **** (*p* < 0.0001). There was also a significant difference between WT mice with and without MI in the suboptimal B2 group—as indicated by ^##^ (*p* = 0.007). In addition, there was a significant difference between WT mice with suboptimal B2 and vitamin mix in the absence of MI—indicated by ^^ (*p* = 0.006); (**C**) Perirenal fat deposition at GD18.5 expressed as percentage of total body weight (%). WT mice in the suboptimal B2 group had less perirenal fat when supplemented with MI—as indicated by ^##^ (*p* = 0.009). In addition, WT mice on suboptimal B2 had more perirenal fat than those on vitamin mix, in the absence of MI, ^^ (*p* = 0.022). In the presence of MI, WT mice had more perirenal fat in the normal B2 group than the vitamin mix group, ^ (*p* = 0.023). In db/+ mice, those in the suboptimal B2 group had more perirenal fat than those on normal B2, in the presence of MI, ^^ (*p* = 0.002). *n* = 7–12 mice per group.

The db/+ mice had greater retroperitoneal fat deposition than WT mice (*p* < 0.0001; Figure 5A).

There was no significant three-way interaction between genotype, MI, and vitamin status on retroperitoneal fat deposition at GD18.5 (*p* = 0.788). There were also no significant two-way interactions or main effects.

#### 3.6.2. Gonadal Fat

The db/+ mice had increased gonadal fat deposition compared to WT mice (*p* = 0.0001; Figure 5B).

There was a three-way interaction between genotype, MI, and vitamin status on gonadal fat deposition at GD18.5 (*p* = 0.031), as a result of a simple two-way interaction between MI and B2 for WT mice (*p* = 0.007), but not for db/+ mice (*p* = 0.179).

*Effect of myo-inositol status*: MI supplementation was associated with reduced gonadal fat deposition in WT mice receiving suboptimal B2 (*p* = 0.007).

*Effect of vitamin status*: Supplementation with vitamin mix resulted in reduced gonadal fat deposition compared with mice receiving suboptimal B2 (*p* = 0.006). This was only observed in WT mice not supplemented with MI.

#### 3.6.3. Perirenal Fat

There was a three-way interaction between genotype, MI, and vitamin status on perirenal fat deposition (*p* = 0.006; Figure 5C), as a result of a simple two-way interaction between MI and vitamin status in WT mice (*p* = 0.026).

*Effect of myo-inositol status*: In WT mice, supplementation with MI reduced perirenal fat deposition in the suboptimal B2 group (*p* = 0.009).

*Effect of vitamin status*: In the absence of MI, WT mice in the vitamin mix group had reduced perirenal fat deposition compared to mice on suboptimal B2 (*p* = 0.022). In the presence of MI, WT mice in the vitamin mix group had reduced perirenal fat deposition compared to mice in the normal B2 group (*p* = 0.023). In db/+ mice supplemented with MI, mice on suboptimal B2 also had increased perirenal fat compared to mice on normal B2 (*p* = 0.002).

### 3.7. Foetal Growth at Day 18.5 of Pregnancy

Foetal growth data are provided in Tables S2 and S3. There were no effects of genotype, and no consistent effects of the supplements, on foetal growth. 

### 3.8. Gene Expression in Adipose Tissue at Day 18.5 of Pregnancy

Gene expression data are presented in Table S4.

#### 3.8.1. Insulin and Leptin Signalling

(a) Akt2: There were no significant three- or two-way interactions between the groups on Akt2 expression. There was a main effect of vitamin status, whereby mice on normal B2 had higher expression of Akt2 than mice on vitamin mix (*p* = 0.010).
(b) Igf1R: There were no significant three- or two-way interactions between the groups on Igf1R expression. There was a main effect of MI status, whereby mice on MI supplementation had higher expression of Igf1R (*p* = 0.020). There was also a main effect of vitamin status, whereby mice in the normal B2 group had decreased Igf1R expression compared to those in both the suboptimal B2 group (*p* = 0.025) and the supplemented B2 group (*p* = 0.019).
(c) LepR: There was a three-way interaction between genotype, MI, and vitamin status (*p* = 0.023), as a result of a simple two-way interaction between MI and vitamin status in db/+ mice (*p* = 0.028). Within the db/+ mice that were supplemented with B2, those not receiving MI had increased LepR expression compared with those receiving MI (*p* = 0.014). Within the db/+ mice that were not treated with MI, those on supplemented B2 had higher LepR expression than mice on both suboptimal B2 (*p* = 0.005) and mice on normal B2 (*p* = 0.010).
(d) IRS1: There were no main effects or interactions between any of the groups on IRS1 expression.
(e) GLUT4: There were no significant three- or two-way interactions between the groups on GLUT4 expression. There was a main effect of vitamin status, whereby mice on normal B2 had higher GLUT4 expression than mice on both suboptimal B2 (*p* = 0.049) and mice on vitamin mix (*p* = 0.015).

#### 3.8.2. Lipid Metabolism

(f) Acsl1: There were no main effects or interactions between any of the groups on Acsl1 expression.

#### 3.8.3. Inflammatory Markers

(g) IL-1β: There were no significant three- or two-way interactions between the groups on IL-1β expression. There was a main effect of vitamin status, whereby mice on normal B2 had higher IL-1β expression than those on both supplemented B2 (*p* = 0.035) and vitamin mix (*p* = 0.026).
(h) Tlr4: There was no significant three-way interaction between genotype, MI, and vitamin status. There was, however, a significant two-way interaction between MI and vitamin status. Within the mice supplemented with MI, those on supplemented B2 (*p* = 0.013) and vitamin mix (*p* = 0.015) had reduced Tlr4 expression compared with those on normal B2.
(i) Tnfrsf1b: There were no main effects or interactions between any of the groups on Tnfrsf1b expression.
(j) Ccr5: There was a three-way interaction between genotype, MI, and vitamin status (*p* = 0.049), due to a simple two-way interaction between MI and vitamin status in WT mice (*p* = 0.001). Within the WT mice on normal B2, those receiving MI had greater Ccr5 expression than without MI (*p* = 0.001). In contrast, Ccr5 expression was reduced when WT mice on suboptimal B2 were supplemented with MI (*p* = 0.014). Within WT mice not receiving MI, those on suboptimal B2 had higher Ccr5 expression than those on normal B2 (*p* = 0.002), supplemented B2 (*p* = 0.016), and vitamin mix (*p* = 0.001). However, in the presence of MI, there was only a difference between normal B2 and supplemented B2—whereby those on supplemented B2 had reduced Ccr5 expression compared to those mice on normal B2 (*p* = 0.014).

There was also an effect of B2 in db/+ mice: within db/+ mice on MI, those on normal B2 had increased Ccr5 expression compared with those on supplemented B2 (*p* = 0.025).

(k) Nlrp3: db/+ mice had increased Nlrp3 expression compared to WT mice (*p* = 0.012). There were no significant three- or two-way interactions between the groups on Nlrp3 expression.

#### 3.8.4. Glucose Metabolism

(l) G6pc: There were no significant three- or two-way interactions between the groups on G6pc expression. There was a main effect of MI, whereby those mice supplemented with MI had lower expression of G6pc than those not on MI (*p* = 0.023).
(m) Gck: There was no significant three-way interaction between genotype, MI, and vitamin status. There was, however, a significant two-way interaction between MI and vitamin status (*p* = 0.018). Within the mice on normal B2, those receiving MI had increased glucokinase expression (*p* < 0.0001). This expression was greater than that seen in mice on both suboptimal (*p* = 0.016) and supplemented B2 (*p* = 0.017).
(n) Gys1: There were no main effects or interactions between any of the groups on Gys1 expression.
(o) Pck1: There were no significant three- or two-way interactions between the groups on Pck1 expression. There was a main effect of MI, whereby those mice on MI had higher expression of Pck1 (*p* = 0.043). There was also a main effect of vitamin status, whereby mice on normal B2 had higher expression of Pck1 than those on supplemented B2 (*p* = 0.003).

#### 3.8.5. Apoptotic Marker

(p) Fas: There was no significant three-way interaction between genotype, MI, and vitamin status on Fas expression. There was, however, a significant two-way interaction between MI and vitamin status (*p* = 0.047). In the suboptimal B2 group, mice receiving MI had lower expression of Fas compared to those not supplemented with MI (*p* = 0.045). Within those mice not receiving MI, those on vitamin mix had lower expression of Fas than those in the normal B2 (mean difference of 0.403 (*p* = 0.031) and suboptimal B2 groups (*p* = 0.001).

## 4. Discussion

This study examined the effects of dietary supplementation of MI and B and D vitamins, individually and in combination, on a mouse model of GDM. GDM has become increasingly common, and is associated with poor maternal and fetal outcomes, both in the short- and long-term. It has been hypothesised that taking MI and B and D vitamins before and/or during pregnancy could counter the negative effects of GDM.

### 4.1. Suitability of db/+ Mice as a Model of GDM

In the current study, db/+ mice did not demonstrate impaired glucose tolerance compared to WT mice during pregnancy. In addition, while db/+ mice gained more weight prior to pregnancy, they did not differ in food consumption or weight gained during pregnancy. These observations are at odds with the majority of other studies in this strain of mouse, where db/+ mice present with hyperphagia, hyperleptinemia, hyperglycaemia, and glucose intolerance during, but not prior to, pregnancy [56,57,64,65,66]. However, several recent studies have similarly been unable to reproduce glucose intolerance and pregnancy weight gain in these animals [67,68]. While db/+ mice have been used as a model of GDM for decades [59], it seems that, for at least the past few years, the glucose intolerance phenotype has been absent in some colonies. Reasons for this may include diet, strain, environmental factors [69], and the absence of the misty (m) allele—which was bred out of the Jackson 000697 strain in 2008 [70]. Our lab is conducting further independent research in order to determine the cause of the lack of glucose intolerance in this model, with the aim of restoring the phenotype for future GDM studies.

However, the db/+ mice in this study still presented with several characteristics of GDM. db/+ mice displayed higher body weight at GD0.5 and GD18.5, but this did not influence OGTT results either prior to pregnancy (data not included) or during pregnancy. In addition, db/+ mice had increased retroperitoneal and gonadal fat deposition, and increased fasting leptin concentrations compared to WT mice. Hyperleptinemia during pregnancy is associated with GDM [71,72,73]. db/+ mice also showed increased gene expression of Nlrp3, a key component of the IL-1β processing NLRP3 inflammasome, in gonadal adipose tissue. It is likely that this increased expression of Nlrp3 was a direct result of the excess fat deposition in db/+ mice. Nlrp3 is part of a family of innate immune cell sensors, which act to recognise danger associated molecular patterns (DAMPS) derived from injured or damaged cells, and subsequently activate an inflammatory response [74,75]. Nlrp3 is thought to be part of the mechanism by which obesity leads to other pathologies such as T2DM and GDM [76]. Mice deficient in Nlrp3 show improved insulin sensitivity even in the presence of a high fat diet, and weight loss is highly associated with reduced adipose tissue expression of Nlrp3 in both animals and humans [77,78,79]. Interestingly, there was no main effect of genotype in any of the other 15 genes studied. Nevertheless, hyperleptinemia, increased body weight at GD0.5 and GD18.5, increased fat deposition, and increased Nlrp3 expression are all conditions that are relevant to the pathogenesis of GDM. Therefore, we believe the model is still of value in this study.

### 4.2. Effects of Myo-Insoitol

MI significantly reduced the weight of WT mice on suboptimal B2 at GD0.5, and this was accompanied by significant decreases in gonadal and perirenal fat deposition. MI also significantly reduced fasting leptin concentrations in db/+ mice fed a diet supplemented with vitamin B2. The fact that there were no differences in maternal weight gain (independent of differences in litter size), food intake, or fat deposition in this group, suggests that the reduction was due to improved leptin signalling. Gonadal gene expression analysis revealed that MI was actually associated with reduced LepR expression within those db/+ mice on supplemented B2, but that supplemented B2 resulted in increased LepR expression compared to the normal B2 and vitamin mix groups. This suggests that vitamin B2 upregulated expression of LepR, while MI acted downstream of the leptin receptor to improve function. This is possible as MI is involved in most eukaryotic cell signalling pathways. It forms the structural basis of inositol phosphates, which act as a number of second messengers, and therefore propagates leptin signalling [80,81,82].

MI supplementation was also associated with increased gonadal adipose tissue gene expression of Igf1R, Pck1, and Gck (glucokinase), and decreased expression of G6pc. Increased Igf1R expression in adipose tissue is associated with reduced incidence of diabetes, because it promotes insulin signalling and therefore favours fat storage over ectopic energy storage [83]. Similarly, increased adipose-specific Pck1 expression is associated with improved insulin sensitivity, due to increased glyceroneogenesis and therefore reduced release of fatty acids into the blood [84]. Gck (glucokinase) catalyses the phosphorylation of glucose to glucose-6-phosphate, and therefore acts to improve glucose uptake within adipose tissue [85]. Finally, G6pc (glucose-6-phosphatase) hydrolyses glucose-6-phosphate, for the final step of both gluconeogenesis and glycogenolysis. Therefore, a reduction in expression of G6pc is associated with reduced release of glucose from adipose tissue, and improved adipose energy storage [86]. These results suggest that MI improved insulin sensitivity of adipose tissue, increased adipose tissue capacity and prevented lipid breakdown and subsequent ectopic energy storage (such as what occurs in non-alcoholic fatty liver disease [87]).

MI supplementation was also associated with improved inflammatory profile, namely reduced expression of Fas, and Ccr5. Fas and Ccr5 are both indicators of cell death and inflammation, demonstrating the anti-inflammatory properties of MI [88,89]. In addition, Ccr5 has been implicated in the development of insulin resistance, which further illustrates the role of MI in improved insulin sensitivity [90]. 

### 4.3. Effects of Suboptimal B2

Mice on the suboptimal B2 diet were significantly heavier at GD0.5 and had significantly increased gonadal and perirenal fat deposition at GD18.5 compared to mice on the normal B2 diet. Low intake of vitamin B2 is associated with increased adiposity, although causation has not been established [91]. The current study therefore points to a causative effect of vitamin B2 in the development of obesity. Vitamin B2 possibly exerts such effects through its role in DNA methylation [92]. For example, human studies have revealed that methylation of the HIF3A locus is associated with BMI [25,93].

Suboptimal B2 was also associated with decreased gonadal fat gene expression of the glucose transporter GLUT4 and Gck, and increased expression of the inflammatory marker Ccr5. These findings are consistent with evidence that vitamin B2 consumption is beneficial for improved insulin sensitivity and dampened inflammation [94,95,96].

### 4.4. Effects of Supplemented B2

B2 supplementation alone had very few effects on the mice in the current study. As stated above, the combination of supplemented B2 and MI resulted in a marked reduction in plasma leptin concentration. This was accompanied by an increase in LepR expression in those mice supplemented with B2. While limited, there is some evidence that vitamin B2 supplementation has a beneficial effect on circulating leptin concentration in obese rats [97] Interestingly, however, B2 supplementation alone did not reduce plasma leptin concentration, and actually increased it compared to mice on suboptimal B2 (in the absence of MI). This occurred despite increased LepR expression in those mice supplemented with B2. The combination of these findings suggest that the increase in LepR expression in mice supplemented with B2 was perhaps in response to hyperleptinemia, and that MI was required in order to assist in the propagation of increased leptin receptor signalling.

B2 supplementation also resulted in a reduction in IL-1β Tlr4, and Ccr5 expression, which again demonstrates the beneficial effects of vitamin B2 on inflammation. In contrast, while supplemented B2 was associated with an increase in Igf1R expression, it also resulted in a reduction in Gck and Pck1 expression. These findings may explain why B2 alone had few beneficial physiological effects on the mice, and why MI co-supplementation was required in order to see differences. The combination of supplemented B2 and MI did appear to generate the most beneficial effects, even if these were not always statistically significant. For example, db/+ mice given the combination of MI and supplemented B2 had the lowest numerical mean value of all the groups in fasting glucose, HOMA-IR, leptin, and gonadal fat deposition.

### 4.5. Effects of Vitamin Mix (B2, B6, B12, and D)

Interestingly, the vitamin mix diet did not have the same effect on plasma leptin that B2 supplementation did, even in the presence of MI. WT mice on the vitamin mix did have decreased gonadal and perirenal fat deposition compared to mice on normal B2, but the same effect was not observed in db/+ mice. As with the B2 supplemented group, vitamin mix was associated with reduced IL-1β, Tlr4, and Fas expression. This again points to the beneficial effects of increased vitamin status on adipose tissue inflammation. The lack of significant differences between the vitamin mix group and the other groups may simply suggest that the mice already had sufficient dietary levels of the vitamins. There are still no established causative effects of excess amounts of these vitamins on improved maternal metabolic health [98,99].

## 5. Conclusions

Strengths of this study include the factorial design, which allowed the examination of the effects of MI in four different vitamin environments, including the individual effect of vitamin B2. This design also allowed us to employ a three-way ANOVA, which meant that groups that were not significantly different could be pooled for added statistical power. The major weakness of this study was that our mouse model—the db/+ mouse—did not exhibit the expected phenotype of glucose intolerance and insulin resistance during pregnancy. It would be beneficial to repeat these experiments in an animal model that does show these properties, since they are the primary characteristics of GDM. However, the db/+ mice in the current study did have increased body weight, fat deposition, and fasting plasma leptin concentrations, along with increased Nlrp3 expression. All of these have been associated with GDM, and so we believe the model is still of value. However, we would recommend that researchers ensure the glucose intolerance phenotype is present before using db/+ mice in the future, as there is evidence that this phenotype is no longer present in some colonies.

Our data demonstrated that suboptimal B2 indeed resulted in unfavourable outcomes, including increased body weight and fat deposition, decreased GLUT4 gene expression, and increased expression of inflammatory markers. MI tended to negate these issues, including reducing body weight and fat deposition, and reducing the expression of inflammatory markers. MI also significantly reduced the marked hyperleptinemia observed in db/+ mice, when combined with supplemented B2. Adipose gene expression data demonstrated that MI was generally associated with markers of improved insulin sensitivity and glucose uptake, and reduced release of glucose and free fatty acids into the blood.

Supplemented B and D vitamins had few physiological effects, although gene expression analysis revealed a reduction in all markers of inflammation that were studied.

In conclusion, both vitamin B2 and MI showed promise in reducing the pathologies associated with GDM in this study. In particular, MI and vitamin B2 were beneficial in reducing body weight and fat deposition, and in improving hyperleptinemia. MI was also effective in improving gene expression markers of insulin sensitivity and glucose uptake, and vitamins B2, B6, B12, and D were associated with a reduction in gene expression of inflammatory markers. Dietary supplementation of both MI and vitamin B2 before and during pregnancy may be an appealing strategy for preventing GDM, but should first be further explored in more relevant animal models.

## Figures and Tables

**Table 1:** Amounts of micronutrients of interest in each of the eight diets, per kg.

Diet	MI (myo-inositol)	B2	B6, B12, D
Suboptimal B2 (−B2)	-	1 mg	7 mg, 25 μg, 1000 IU
Suboptimal B2 + MI (−B2 + MI)	10 g	1 mg	7 mg, 25 μg, 1000 IU
AIN-93G (B2)	-	6 mg	7 mg, 25 μg, 1000 IU
MI only (B2 + MI)	10 g	6 mg	7 mg, 25 μg, 1000 IU
B2 only (+B2)	-	24 mg	7 mg, 25 μg, 1000 IU
B2 + MI (+B2 + MI)	10 g	24 mg	7 mg, 25 μg, 1000 IU
Vitamin Mix (VM)	-	24 mg	28 mg, 215 μg, 4000 IU
MI + vitamin mix (VM + MI)	10 g	24 mg	28 mg, 215 μg, 4000 IU

## Supplementary Materials

The following are available online at http://www.mdpi.com/2072-6643/9/2/141/s1, Figure S1: Glucose tolerance at GD16.5 of WT and db/+ mice in the control group (normal B2, no added MI). (A) Oral glucose tolerance plots comparing WT and db/+ mice on control diet. There were no differences between WT and db/+ mice on control diet at any time point of the OGTT; (B) The area under the curve of the glucose tolerance plots comparing WT and db/+ mice on control diet. There was no difference between WT and db/+ mice on area under the curve of the OGTT plots. *n* = 12 mice per group; Figure S2: Glucose tolerance at GD16.5, when supplement groups are pooled together for WT and db/+ mice: (A) Oral glucose tolerance plots comparing all WT and all db/+ mice. There were no differences between WT and db/+ mice when all supplements were pooled at any time point of the OGTT. (B) The area under the curve of the glucose tolerance plots comparing all WT and all db/+ mice. There was no difference between WT and db/+ mice on area under the curve of the OGTT plots; Table S1: Weights of major organs at GD18.5. Data are presented as the mean ± SEM; Table S2: Fetal and placental growth data from WT mothers; Table S3: Fetal and placental growth data from db/+ mothers; Table S4: Gene expression relative to db/+ mice (on normal B2, no MI), of 16 genes examined in gonadal adipose tissue.
