# Supplementary material for: The Effects of Myo-Inositol and B and D Vitamin Supplementation in the db/+ Mouse Model of Gestational Diabetes Mellitus
Source: Nutrients. 2017 Feb 15;9(2):141. doi: 10.3390/nu9020141 (PMC5331572; doi:10.3390/nu9020141)
Supplement: Supplementary file 1 [file nutrients-09-00141-s001.docx]

Supplementary Materials

Genotyping procedure.

Tail samples were incubated in 75 μL of 25 mM NaOH/0.2 mM EDTA at 95 °C for 1 h. 75 μL of 40 mM Tris HCl (pH 5.5) was then added, and the final mix was left at −20 °C overnight. DNA was amplified using 12 μL KAPA HotStart ReadyMix with dye (KAPA BioSystems, Wilmington, MA, USA), 1.2 μL forward primer (Integrated DNA Technologies, Coralville, IA, USA; 5’—AGA ACG GAC ACT CTT TGA AGT CTC—3’), 1.2 μL reverse primer (Integrated DNA Technologies, IA, USA; 5’—CAT TCA AAC CAT AGT TTA GGT TTG TGT—3’) and 8.6 μL H_2_O per reaction. Amplification was performed at 1 cycle of 95 °C for 3 min, 32 cycles of 95 °C for 15 s, 60 °C for 15 s, 72 °C for 15 s, and 1 cycle at 72 °C for 1 min. The reaction was then left to incubate for 18 h with 1 μL restriction enzyme RsaI (NewEngland Biolabs, Ipswich, MA, USA), 5 μL CutSmart buffer, and 19 ΜL H_2_O per reaction at 35 °C. Products were resolved on a 4% agarose gel and visualised with Ethidium Bromide. The presence of a band at 135 bp indicated that the mouse was a wild-type (WT), while two bands at 108 bp and 135 bp indicated a heterozygote (db/+).


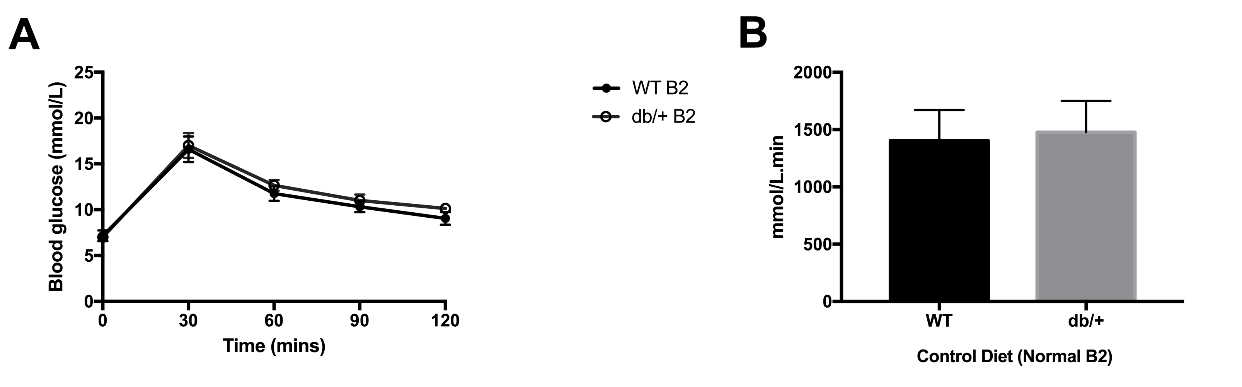


**Figure S1.** Glucose tolerance at GD16.5 of WT and db/+ mice in the control group (normal B2, no added MI). (**A**) Oral glucose tolerance plots comparing WT and db/+ mice on control diet. There were no differences between WT and db/+ mice on control diet at any time point of the OGTT; (**B**) The area under the curve of the glucose tolerance plots comparing WT and db/+ mice on control diet. There was no difference between WT and db/+ mice on area under the curve of the OGTT plots. *n* = 12 mice per group.


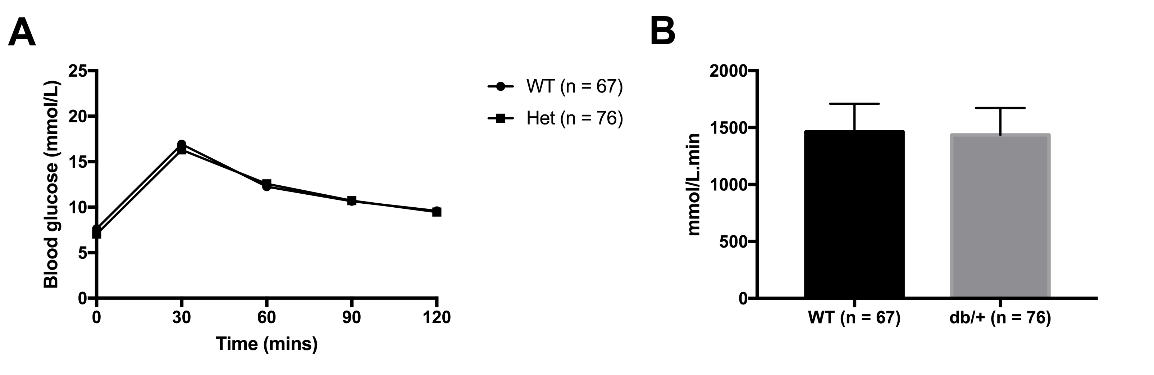


**Figure S2.** Glucose tolerance at GD16.5, when supplement groups are pooled together for WT and db/+ mice. (**A**) Oral glucose tolerance plots comparing all WT and all db/+ mice. There were no differences between WT and db/+ mice when all supplements were pooled at any time point of the OGTT; (**B**) The area under the curve of the glucose tolerance plots comparing all WT and all db/+ mice. There was no difference between WT and db/+ mice on area under the curve of the OGTT plots.

**Table S1.** Weights of major organs at GD18.5. Data is presented as the mean ± SEM.

| ***Genotype*** | **WT** | | | | | | | |
| --- | --- | --- | --- | --- | --- | --- | --- | --- |
| ***MI Status*** | **− MI** | | | | **+ MI** | | | |
| ***B Vitamin status*** | **−B2** | **B2** | **+B2** | **VM** | **−B2** | **B2** | **+B2** | **VM** |
| Pancreas Weight (g) | 0.29 ± 0.04 | 0.26 ± 0.03 | 0.25 ± 0.04 | 0.20 ± 0.04 | 0.23 ± 0.04 | 0.23 ± 0.04 | 0.23 ± 0.04 | 0.22 ± 0.04 |
| Spleen Weight (g) | 0.21 ± 0.03 | 0.20 ± 0.02 | 0.19 ± 0.03 | 0.16 ± 0.03 | 0.21 ± 0.03 | 0.22 ± 0.03 | 0.18 ± 0.03 | 0.21 ± 0.03 |
| Kidney weight (g) | 0.18 ± 0.01 | 0.17 ± 0.01 | 0.16 ± 0.01 | 0.16 ± 0.01 | 0.17 ± 0.01 | 0.18 ± 0.01 | 0.18 ± 0.01 | 0.18 ± 0.01 |
| Liver Weight (g) | 1.31 ± 0.08 | 1.32 ± 0.07 | 1.34 ± 0.08 | 1.50 ± 0.08 | 1.26 ± 0.08 | 1.23 ± 0.08 | 1.36 ± 0.09 | 1.42 ± 0.09 |
| ***Genotype*** | **db/+** | | | | | | | |
| ***MI status*** | **− MI** | | | | **+ MI** | | | |
| ***B Vitamin status*** | **−B2** | **B2** | **+B2** | **VM** | **−B2** | **B2** | **+B2** | **VM** |
| Pancreas Weight (g) | 0.25 ± 0.03 | 0.27 ± 0.03 | 0.27 ± 0.03 | 0.26 ± 0.04 | 0.26 ± 0.03 | 0.23 ± 0.03 | 0.27 ± 0.04 | 0.26 ± 0.03 |
| Spleen Weight (g) | 0.20 ± 0.03 | 0.21 ± 0.02 | 0.19 ± 0.03 | 0.17 ± 0.03 | 0.21 ± 0.03 | 0.24 ± 0.03 | 0.23 ± 0.03 | 0.28 ± 0.03 |
| Kidney weight (g) | 0.17 ± 0.01 | 0.18 ± 0.01 | 0.18 ± 0.01 | 0.18 ± 0.01 | 0.16 ± 0.01 | 0.20 ± 0.01 | 0.18 ± 0.01 | 0.18 ± 0.01 |
| Liver Weight (g) | 1.41 ± 0.08 | 1.40 ± 0.07 | 1.39 ± 0.07 | 1.66 ± 0.09 | 1.35 ± 0.08 | 1.21 ± 0.08 | 1.39 ± 0.08 | 1.34 ± 0.07 |

**Table S2.** Fetal and placental growth data from WT mothers.

| ***MI Status*** | **− MI** | | | | **+ MI** | | | |
| --- | --- | --- | --- | --- | --- | --- | --- | --- |
| ***Vitamin Status*** | **−B2** | **B2** | **+B2** | **VM** | **−B2** | **B2** | **+B2** | **VM** |
| Fetal Weight (g) | 1.14 ± 0.05 | 1.11 ± 0.05 | 1.15 ± 0.05 | 1.11 ± 0.02 | 1.14 ± 0.03 | 1.09 ± 0.05 | 1.11 ± 0.003 | 1.06 ± 0.09 |
| Crown-Rump Length (mm) | 30.41 ± 0.42 | 30.41 ± 0.42 | 30.13 ± 0.54 | 30.59 ± 0.17 | 29.66 ± 0.59 | 29.66 ± 0.59 | 30.10 ± 0.47 | 29.69 ± 0.84 |
| Abdominal Circumference (mm) | 25.57 ± 0.69 | 25.09 ± 0.36 | 25.06 ± 0.55 | 25.00 ± 0.52 | 25.96 ± 0.29 | 25.16 ± 0.41 | 25.53 ± 0.50 | 25.27 ± 1.31 |
| Placental Weight (g) | 0.10 ± 0.01 | 0.09 ± 0.00 | 0.11 ± 0.01 | 0.09 ± 0.01 | 0.11 ± 0.01 | 0.09 ± 0.00 | 0.09 ± 0.01 | 0.11 ± 0.01 |
| Fetal : Placental Ratio | 10.82 ± 0.77 | 12.26 ± 0.63 | 11.39 ± 0.88 | 11.96 ± 0.79 | 10.81 ± 0.72 | 11.89 ± 0.41 | 12.76 ± 0.92 | 10.11 ± 1.12 |

**Table S3.** Fetal and placental growth data from db/+ mothers.

| ***Pup Genotype*** | **WT Pups** | | | | | | | |
| --- | --- | --- | --- | --- | --- | --- | --- | --- |
| ***MI Status*** | **− MI** | | | | **+ MI** | | | |
| ***Vitamin Status*** | **−B2** | **B2** | **+B2** | **VM** | **−B2** | **B2** | **+B2** | **VM** |
| Fetal Weight (g) | 1.13 ± 0.04 | 1.13 ± 0.04 | 1.18 ± 0.10 | 1.10 ± 0.05 | 1.15 ± 0.04 | 0.93 ± 0.07 | 1.10 ± 0.05 | 1.09 ± 0.04 |
| Crown-Rump Length (mm) | 30.32 ± 0.51 | 30.63 ± 0.40 | 30.38 ± 0.80 | 29.81 ± 0.57 | 30.59 ± 0.44 | 28.93 ± 0.72 | 29.93 ± 0.51 | 30.69 ± 0.39 |
| Abdominal Circumference (mm) | 25.96 ± 0.47 | 24.96 ± 0.39 | 26.75 ± 0.77 | 25.31 ± 0.55 | 25.44 ± 0.45 | 24.00 ± 0.69 | 25.71 ± 0.49 | 25.39 ± 0.38 |
| Placental Weight (g) | 0.09 ± 0.01 | 0.09 ± 0.00 | 0.09 ± 0.01 | 0.09 ± 0.01 | 0.09 ± 0.00 | 0.10 ± 0.01 | 0.10 ± 0.01 | 0.09 ± 0.00 |
| Fetal : Placental Ratio | 11.61 ± 0.78 | 12.63 ± 0.59 | 13.13 ± 1.18 | 11.89 ± 0.83 | 12.55 ± 0.68 | 9.44 ± 1.05 | 10.86 ± 0.74 | 12.88 ± 0.55 |
| ***Genotype*** | **db/+ Pups** | | | | | | | |
| ***MI Status*** | **− MI** | | | | **+ MI** | | | |
| ***Vitamin Status*** | **−B2** | **B2** | **+B2** | **VM** | **−B2** | **B2** | **+B2** | **VM** |
| Fetal Weight (g) | 1.08 ± 0.04 | 1.13 ± 0.03 | 1.18 ± 0.05 | 1.04 ± 0.04 | 1.17 ± 0.05 | 1.10 ± 0.05 | 1.08 ± 0.04 | 1.09 ± 0.03 |
| Crown-Rump Length (mm) | 29.87 ± 0.48 | 30.68 ± 0.36 | 30.92 ± 0.57 | 29.72 ± 0.46 | 31.10 ± 0.51 | 30.72 ± 0.48 | 29.60 ± 0.44 | 29.93 ± 0.36 |
| Abdominal Circumference (mm) | 25.17 ± 0.44 | 25.10 ± 0.35 | 25.71 ± 0.54 | 25.38 ± 0.44 | 25.90 ± 0.49 | 24.98 ± 0.47 | 25.16 ± 0.41 | 24.41 ± 0.35 |
| Placental Weight (g) | 0.10 ± 0.01 | 0.10 ± 0.00 | 0.10 ± 0.01 | 0.09 ± 0.00 | 0.09 ± 0.01 | 0.10 ± 0.01 | 0.10 ± 0.00 | 0.09 ± 0.00 |
| Fetal: Placental Ratio | 11.30 ± 0.71 | 11.75 ± 0.54 | 11.93 ± 0.83 | 11.40 ± 0.71 | 13.17 ± 0.74 | 10.97 ± 0.71 | 11.14 ± 0.65 | 12.03 ± 0.54 |
| ***Genotype*** | **db/db Pups** | | | | | | | |
| ***MI Status*** | **− MI** | | | | **+ MI** | | | |
| ***Vitamin Status*** | **−B2** | **B2** | **+B2** | **VM** | **−B2** | **B2** | **+B2** | **VM** |
| Fetal Weight (g) | 1.13 ± 0.06 | 1.23 ± 0.06 | 1.28 ± 0.07 | 1.06 ± 0.04 | 1.22 ± 0.06 | 1.19 ± 0.06 | 1.18 ± 0.07 | 1.08 ± 0.05 |
| Crown-Rump Length (mm) | 28.93 ± 0.60 | 31.13 ± 0.80 | 30.33 ± 0.92 | 29.31 ± 0.51 | 31.17 ± 0.80 | 30.89 ± 0.65 | 28.50 ± 1.60 | 30.00 ± 0.72 |
| Abdominal Circumference (mm) | 25.44 ± 0.55 | 25.25 ± 0.77 | 25.67 ± 0.89 | 26.28 ± 0.49 | 25.88 ± 0.77 | 26.10 ± 0.63 | 22.00 ± 1.54 | 24.67 ± 0.63 |
| Placental Weight (g) | 0.10 ± 0.01 | 0.09 ± 0.01 | 0.10 ± 0.01 | 0.09 ± 0.01 | 0.10 ± 0.01 | 0.10 ± 0.01 | 0.11 ± 0.02 | 0.09 ± 0.01 |
| Fetal : Placental Ratio | 11.32 ± 0.89 | 13.58 ± 1.18 | 11.58 ± 1.36 | 11.52 ± 0.74 | 12.61 ± 1.18 | 11.58 ± 0.96 | 8.74 ± 2.35 | 11.89 ± 0.96 |

**Table S4.** Gene expression relative to db/+ mice (on normal B2, no MI), of 16 genes examined in gonadal adipose tissue.

| ***Genotype*** | **WT** | | | | | | | |
| --- | --- | --- | --- | --- | --- | --- | --- | --- |
| ***MI Status*** | **− MI** | | | | **+ MI** | | | |
| ***Vitamin Status*** | **−B2** | **B2** | **+B2** | **VM** | **−B2** | **B2** | **+B2** | **VM** |
| *Insulin and leptin signalling* | |  |  |  |  |  |  |  |
| Akt2 * | 0.628 ± 0.168 | 0.603 ± 0.137 | 0.858 ± 0.168 | 0.654 ± 0.150 | 0.845 ± 0.168 | 1.145 ± 0.168 | 0.526 ± 0.168 | 0.425 ± 0.194 |
| Igf1R * | 1.000 ± 0.759 | 1.148 ± 0.537 | 2.253 ± 0.759 | 1.294 ± 0.679 | 3.241 ± 0.759 | 1.472 ± 0.759 | 3.530 ± 0.679 | 2.507 ± 0.876 |
| LepR | 1.086 ± 0.423 | 1.809 ± 0.299 | 1.526 ± 0.423 | 1.406 ± 0.378 | 2.086 ± 0.423 | 1.410 ± 0.423 | 2.840 ± 0.378 | 2.728 ± 0.489 |
| IRS-1 | 0.660 ± 0.183 | 0.823 ± 0.130 | 0.822 ± 0.183 | 0.568 ± 0.164 | 0.755 ± 0.183 | 0.792 ± 0.183 | 0.760 ± 0.164 | 0.590 ± 0.212 |
| GLUT4 * | 1.027 ± 0.276 | 0.896 ± 0.195 | 0.802 ± 0.276 | 0.388 ± 0.247 | 0.309 ± 0.276 | 1.601 ± 0.276 | 0.454 ± 0.247 | 0.798 ± 0.319 |
| *Lipid metabolism* | |  |  |  |  |  |  |  |
| Acsl1 | 2.131 ± 0.560 | 2.511 ± 0.396 | 1.715 ± 0.560 | 1.416 ± 0.501 | 1.081 ± 0.560 | 2.576 ± 0.560 | 1.182 ± 0.501 | 0.933 ± 0.647 |
| *Inflammatory markers* | |  |  |  |  |  |  |  |
| IL-1b * | 1.137 ± 0.350 | 1.223 ± 0.248 | 0.340 ± 0.350 | 0.481 ± 0.313 | 0.350 ± 0.350 | 1.109 ± 0.350 | 0.400 ± 0.313 | 0.651 ± 0.405 |
| Tlr4 * | 1.563 ± 0.383 | 1.195 ± 0.271 | 0.917 ± 0.383 | 1.030 ± 0.343 | 1.496 ± 0.383 | 2.749 ± 0.383 | 0.852 ± 0.343 | 0.954 ± 0.443 |
| Tnfrsf1b | 0.711 ± 0.343 | 1.064 ± 0.243 | 1.663 ± 0.343 | 0.948 ± 0.307 | 1.727 ± 0.343 | 0.937 ± 0.343 | 1.417 ± 0.307 | 1.223 ± 0.396 |
| Ccr5 * | 1.428 ± 0.180 | 0.583 ± 0.127 | 0.637 ± 0.180 | 0.480 ± 0.161 | 0.788 ± 0.180 | 1.370 ± 0.180 | 0.608 ± 0.161 | 0.815 ± 0.208 |
| Nlrp3 ^ | 2.081 ± 0.407 | 1.007 ± 0.288 | 1.135 ± 0.407 | 0.957 ± 0.364 | 1.249 ± 0.407 | 1.331 ± 0.407 | 1.019 ± 0.364 | 1.891 ± 0.469 |
| *Glucose metabolism* | |  |  |  |  |  |  |  |
| G6pc * | 1.143 ± 0.345 | 0.405 ± 0.261 | 1.568 ± 0.345 | 0.796 ± 0.308 | 0.830 ± 0.345 | 0.814 ± 0.398 | 0.605 ± 0.308 | 0.951 ± 0.398 |
| Gck * | 0.373 ± 1.670 | 0.613 ± 0.964 | 1.373 ± 1.181 | 2.266 ± 1.056 | 1.131 ± 1.181 | 4.957 ± 1.364 | 1.453 ± 1.181 | 2.012 ± 1.670 |
| Gys1 | 1.214 ± 0.232 | 0.823 ± 0.164 | 0.994 ± 0.232 | 0.855 ± 0.208 | 0.779 ± 0.232 | 1.382 ± 0.232 | 0.739 ± 0.208 | 1.274 ± 0.268 |
| Pck1 * | 0.696 ± 0.182 | 0.853 ± 0.128 | 0.558 ± 0.182 | 0.795 ± 0.162 | 0.754 ± 0.182 | 1.291 ± 0.182 | 0.671 ± 0.162 | 1.230 ± 0.210 |
| *Apoptotic markers* | |  |  |  |  |  |  |  |
| Fas * | 1.383 ± 0.179 | 0.979 ± 0.126 | 0.820 ± 0.179 | 0.470 ± 0.160 | 0.794 ± 0.179 | 1.423 ± 0.179 | 0.635 ± 0.160 | 0.898 ± 0.206 |
| ***Genotype*** | **db/+** | | | | | | | |
| ***MI status*** | **− MI** | | | | **+ MI** | | | |
| ***Vitamin Status*** | **−B2** | **B2** | **+B2** | **VM** | **−B2** | **B2** | **+B2** | **VM** |
| *Insulin and leptin signalling* | |  |  |  |  |  |  |  |
| Akt2 * | 0.784 ± 0.127 | 1.052 ± 0.127 | 0.815 ± 0.137 | 0.660 ± 0.127 | 1.084 ± 0.127 | 0.917 ± 0.150 | 0.836 ± 0.137 | 0.479 ± 0.150 |
| Igf1R * | 2.321 ± 0.574 | 0.887 ± 0.574 | 2.631 ± 0.620 | 3.201 ± 0.574 | 3.959 ± 0.574 | 2.108 ± 0.679 | 2.884 ± 0.620 | 2.023 ± 0.679 |
| LepR * | 1.055 ± 0.320 | 1.161 ± 0.320 | 2.702 ± 0.345 | 1.749 ± 0.320 | 1.801 ± 0.320 | 1.089 ± 0.378 | 1.469 ± 0.345 | 1.122 ± 0.378 |
| IRS-1 | 0.700 ± 0.139 | 1.096 ± 0.150 | 0.559 ± 0.150 | 0.666 ± 0.139 | 0.762 ± 0.139 | 0.920 ± 0.164 | 0.614 ± 0.150 | 0.576 ± 0.164 |
| GLUT4 * | 0.723 ± 0.209 | 1.045 ± 0.209 | 0.882 ± 0.225 | 0.653 ± 0.209 | 0.716 ± 0.209 | 1.074 ± 0.247 | 0.798 ± 0.225 | 0.595 ± 0.247 |
| *Lipid metabolism* | |  |  |  |  |  |  |  |
| Acsl1 | 2.072 ± 0.560 | 0.857 ± 0.423 | 1.536 ± 0.457 | 1.450 ± 0.423 | 2.204 ± 0.423 | 1.684 ± 0.501 | 1.425 ± 0.457 | 1.018 ± 0.501 |
| *Inflammatory markers* | |  |  |  |  |  |  |  |
| IL-1b * | 0.751 ± 0.265 | 1.504 ± 0.265 | 0.874 ± 0.286 | 0.477 ± 0.265 | 0.443 ± 0.265 | 0.740 ± 0.313 | 0.522 ± 0.286 | 0.360 ± 0.313 |
| Tlr4 * | 1.650 ± 0.290 | 1.351 ± 0.290 | 1.532 ± 0.313 | 1.031 ± 0.290 | 1.207 ± 0.290 | 1.687 ± 0.343 | 1.372 ± 0.313 | 1.091 ± 0.343 |
| Tnfrsf1b | 1.485 ± 0.259 | 1.219 ± 0.259 | 1.795 ± 0.280 | 1.538 ± 0.259 | 1.400 ± 0.259 | 1.327 ± 0.307 | 1.402 ± 0.280 | 1.064 ± 0.307 |
| Ccr5 * | 0.863 ± 0.136 | 1.088 ± 0.136 | 0.868 ± 0.147 | 0.556 ± 0.136 | 0.824 ± 0.136 | 1.236 ± 0.161 | 0.590 ± 0.147 | 0.664 ± 0.161 |
| Nlrp3 ^ | 2.452 ± 0.307 | 1.160 ± 0.307 | 1.620 ± 0.332 | 1.595 ± 0.307 | 1.989 ± 0.307 | 1.659 ± 0.364 | 2.034 ± 0.332 | 1.910 ± 0.364 |
| *Glucose metabolism* | |  |  |  |  |  |  |  |
| G6pc * | 1.422 ± 0.282 | 1.186 ± 0.282 | 1.371 ± 0.282 | 1.019 ± 0.261 | 0.558 ± 0.261 | 0.682 ± 0.308 | 1.375 ± 0.282 | 1.147 ± 0.345 |
| Gck * | 4.032 ± 0.964 | 1.099 ± 0.964 | 1.147 ± 0.964 | 1.311 ± 0.893 | 1.687 ± 0.964 | 5.281 ± 1.181 | 1.441 ± 0.964 | 4.025 ± 1.181 |
| Gys1 | 1.175 ± 0.176 | 1.165 ± 0.176 | 0.884 ± 0.190 | 0.927 ± 0.176 | 0.985 ± 0.176 | 1.328 ± 0.208 | 0.801 ± 0.190 | 0.712 ± 0.208 |
| Pck1 * | 1.135 ± 0.137 | 0.991 ± 0.137 | 0.571 ± 0.148 | 0.708 ± 0.137 | 0.976 ± 0.137 | 1.156 ± 0.162 | 0.863 ± 0.148 | 0.698 ± 0.162 |
| *Apoptotic markers* | |  |  |  |  |  |  |  |
| Fas * | 1.043 ± 0.135 | 0.991 ± 0.135 | 0.828 ± 0.146 | 0.695 ± 0.135 | 0.985 ± 0.135 | 0.958 ± 0.160 | 0.627 ± 0.146 | 0.613 ± 0.160 |

An asterix (*) indicates that there were significant effects of treatment for that gene. A ^ indicates that there was a significant effect of genotype for that gene. Please refer to the text for further information on each of these effects. Akt2 = thymoma viral proto-oncognene 2; Igf1R = insulin-like growth factor 1 receptor; LepR = leptin receptor; IRS-1 = insulin receptor substrate 1; GLUT4 = solute carrier family 2 (facilitated glucose transporter), member 4; Acsl1 = acyl-CoA synthetase long-chain family member 1; IL-1b = interleukin 1 beta; Tlr4 = toll-like receptor 4; Tnfrsf1b = tumor necrosis factor receptor superfamily, member 1b; Ccr5 = chemokine receptor 5; Nlrp3 = inflammasome, NLR family, pyrin domain containing 3; G6pc = glucose-6-phosphatase, catalytic; Gck = Glucokinase; Gys1 = glycogen synthase 1; Pck1 = phosphenolpyruvate carboxykinase 1; Fas = cell surface death receptor.
